# Fasting mimicking diet inhibits tumor-associated macrophage survival and pro-tumor function in hypoxia: implications for combination therapy with anti-angiogenic agent

**DOI:** 10.1186/s12967-023-04577-7

**Published:** 2023-10-26

**Authors:** Lei Wang, Yu-jie Wang, Rong Wang, Fu-lian Gong, Yu-huan Shi, Sheng-nan Li, Pan-pan Chen, Yong-fang Yuan

**Affiliations:** 1 Department of Pharmacy, Shanghai 9th People’s Hospital, Shanghai Jiao Tong University School of Medicine, No. 280 Mohe Road, Shanghai, 201999 China; 2 School of Medicine, National Children’s Medical Center, Shanghai Children’s Medical Center, Shanghai Jiao Tong University, No. 1678 Dongfang Road, Shanghai, 200127 China

**Keywords:** Fasting-mimicking diet, Anti-angiogenic drug, Hypoxia, TAMs

## Abstract

**Background:**

Recent research shows that tumor-associated macrophages (TAMs) are the primary consumers of glucose in tumor tissue, surpassing that of tumor cells. Our previous studies revealed that inhibiting glucose uptake impairs the survival and tumor-promoting function of hypoxic TAMs, suggesting that glucose reduction by energy restriction (calorie restriction or short-term fasting) may has a significant impact on TAMs. The purpose of this study is to verify the effect of fasting-mimicking diet (FMD) on TAMs, and to determine whether FMD synergizes with anti-angiogenic drug apatinib via TAMs.

**Methods:**

The effect of FMD on TAMs and its synergistic effects with apatinib were observed using an orthotopic mouse breast cancer model. An in vitro cell model, utilizing M2 macrophages derived from THP-1 cell line, was intended to assess the effects of low glucose on TAMs under hypoxic and normoxic conditions. Bioinformatics was used to screen for potential mechanisms of action, which were then validated both in vivo and in vitro.

**Results:**

FMD significantly inhibit the pro-tumor function of TAMs in vivo and in vitro, with the inhibitory effect being more pronounced under hypoxic conditions. Additionally, the combination of FMD-mediated TAMs inhibition with apatinib results in synergistic anti-tumor activity. This effect is partially mediated by the downregulation of CCL8 expression and secretion by the mTOR-HIF-1α signaling pathway.

**Conclusions:**

These results support further clinical combination studies of FMD and anti-angiogenic therapy as potential anti-tumor strategies.

**Graphical Abstract:**

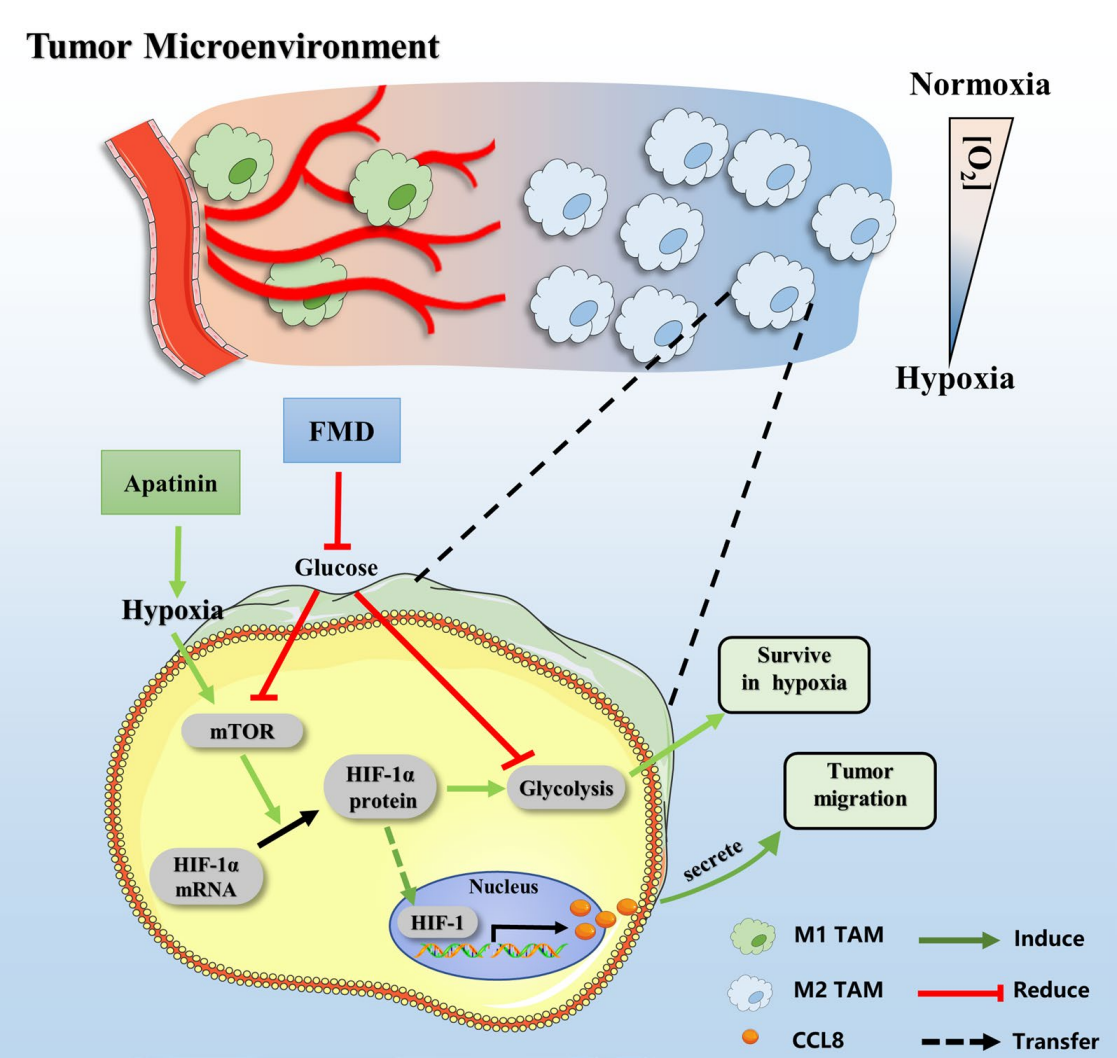

## Introduction

Energy restriction (calorie restriction or short-term fasting) has been investigated as a complementary approach to chemotherapy to reduce the side effects and enhance efficacy of chemotherapy [1–3]. However, long-term caloric restriction or short-term fasting may lead to nutritional deficiencies and is often poorly tolerated by patients, limiting its applicability in the clinic. To overcome this limitation, a fasting-mimicking diet (FMD) has been developed. This diet, which is low in calories and protein yet high in fats, is specifically designed to mimic the effects of fasting while still providing essential nutrients to the body [4]. The safety and feasibility of FMD have been demonstrated in various solid-tumor patients, indicating its potential for use in combination with anti-tumor treatment [5–9]. Nevertheless, the combination of fasting or FMD and anti-angiogenic drugs has not been studied to date.

Recent research has uncovered that anti-angiogenic therapy (AAT) may enhance the hypoxia in tumor, then increase the number of tumor-promoting type tumor-associated macrophages (TAMs) while limiting therapeutic efficacy of AAT [10–12]. TAMs are a diverse population of immune cells that can be categorized into two main functional types, namely M1 and M2. M1 TAMs are predominantly found in well-vascularized tumor areas and have been associated with promising patient prognosis via their ability to hinder tumor cell proliferation and stimulate T cell-mediated antitumor immunity [13, 14]. In contrast, M2 TAMs are primarily located in hypoxic regions and linked to poor prognosis through promoting tumor cell migration, angiogenesis, drug resistance, and immunosuppression [15–18]. As such, selectively targeting TAMs restricted to hypoxic regions without affecting those located in well-vascularized regions represents a promising anti-cancer therapy strategy. Our previous research has found that inhibiting glucose uptake of hypoxic TAMs could reduce its pro-tumor function, then enhanced anti-tumor efficacy of AAT [11, 19]. Clinical and preclinical studies have confirmed that FMD results in reduced glucose levels within tumor tissues [6, 20], suggesting its potential to inhibit the pro-tumor functions of hypoxic TAMs and synergize with AAT.

In our study, we observed the impact of FMD on TAMs and its synergistic effects with apatinib in an orthotopic mouse breast cancer model. Further, we examined the effect of FMD-induced reduced glucose levels on TAMs by investigating the effects of glucose deprivation (GD) on the viability and pro-tumor metastasis function of TAMs in hypoxic environments in vitro. Additionally, bioinformatics was used to screen for potential mechanisms, which were then validated both in vivo and in vitro.

## Materials and methods

### Reagents and materials

WZB117, 2-DG, 2ME2, EVE (MCE, NJ, USA); CoCl_2_ (Sigma, Darmstadt, Germany); liposomal clodronate (YEASEN, Amsterdam, Netherlands); apatinib (Hengrui Corp, Jiangsu, China); anti-human CCL8 (BioLegend, USA).

Lactate acid assay kit (NJJCBIO, Wuhan, China); glucose assay kit (NJJCBIO, Wuhan, China); cell counting kit-8 assay (Meilun, Dalian, China); human and mouse CCL8 mini ABTS ELISA development kit (PeproTech, Hamburg, Germany).

### Cell lines

Human monocyte THP-1, Human breast cancer cell line MDA-MB-231, and mice breast cancer cell line, 4T1, were obtained from Shanghai Institute of Cell Biology (Shanghai, China). All cells were cultured at 37 °C, 5% CO_2_, in RPMI-1640 medium, supplemented with 10% fetal bovine serum (Gibco, CA, USA).

### Polarization of TAMs

Polarization of macrophage was performed as previously described [11, 19]. THP-1 cells were treated for 1 day with 320 nM PMA (Beyotime, Haimen, China), followed by incubation for an additional 3 days with 20 ng/mL of IL-4 and IL-13 (PeproTech, NJ, USA).

### Glucose deprivation

For glucose deprivation experiments, TAMs were exposed to one among the following: (i) control group; (ii) 50% glucose deprivation (GD) control group (50% the glucose concentration of Control group); (iii) 75% GD control group (25% the glucose concentration of Control group).

### Western blotting

Western blot was performed as previously described [11, 19]. Primary antibodies used for western blotting are: HIF-1α, mTOR, p-mTOR, proliferating cell nuclear antigen (pCNA) (Proteintech, China), and anti-β-actin (ZS Bio, China).

### Quantitative real-time PCR (qRT-PCR)

RNA was extracted from the TAMs using the TRIzol method (Invitrogen, California, USA). Total RNA was reversely transcribed into cDNA using One Step PrimeScript™ RT-PCR Kit (TaKaRa, Japan). Target gene sequences were purchased from Boshang Biotechnology (Shanghai, China).

### Transwell co-culture system

TAMs were co-cultured with MDA-MB-231 cells in a transwell chamber (Corning, NY, USA) for 48 h. The MDA-MB-231 cells on the bottom side of the transwell chamber were stained with crystal violet and counted.

### Immunohistochemical staining assay

Immunohistochemical staining assay was performed as previously described [11, 19]. The antibodies used for western blotting are: rabbit anti-mouse F4/80 (1:50, GB113373, BioLegend, Servicebio, Wuhan, China) and anti-mouse CD206 (1:50, GB113497, Servicebio, Wuhan, China) antibodies. Percent area of immunofluorescence staining was quantified using ImageJ (version 1.49p, national institutes of health, USA) normalized by DAPI.

### Fasting-mimicking diet

Two weeks prior to study initiation, the mice were maintained on the rodent standard diet (AIN-93G diet), and the findings were recorded twice per week, to determine baseline of daily energy intake of mice. FMD cycles were designated as ‘Day 1’ and ‘Day 2–3’ diets every week; this diet contains 10% animal-based protein and 90% fat. The ‘Day 1’ and ‘Day 2–3’ diets contain 50% and 10% of the whole energy of baseline feeding, restoring normal diet at other times.

### Breast orthotopic tumor mouse model

BALB/c mice (female, 5 weeks old) were purchased from Vital River Laboratories (Beijing, China). 4T1-Luc cells (5 × 10^5^/100 µL/each mouse) were injected into the mammary fat pad, under #3 mammary gland of each mouse. Once the tumor volume reached 100–200 mm^3^, over-or under-sized mice were excluded. The remaining mice were divided randomly into six groups: control; FMD; apatinib (12.5 mg/kg/day, oral gavage (i.g.)); FMD + apatinib (12.5 mg/kg/day, i.g.); liposomal clodronate (CL) [10 µL/g/week, intravenous (i.v.)]; apatinib (12.5 mg/kg/day, i.g.) + CL (100 µL/10 g/week).

### Kyoto encyclopedia of genes and genomes (KEGG) pathway analysis

KEGG pathway analysis was performed by mapping the KEGG annotated genes to KEGG pathways. KEGG pathway enrichment analysis for alteration of signaling pathway treated with bevacizumab in the dataset GSE37956 was performed with clusterProfiler R package (version 3.12.0) implemented in R version 4.3.0 (ALCATEL-LUCENT USA INC., USA).

### Tumor immune estimation resource (TIMER)

The correlation of CCL8, TGFBI, HGF expression with the abundance of TAM infiltrates in breast cancer was investigated via the publicly available on-line server TIMER (https://cistrome.shinyapps.io/timer/). TAM infiltration in breast cancer treated with everolimus (data from GEO dataset GSE50712, https://www.ncbi.nlm.nih.gov/geo) also were analyzed using the TIMER.

### Gene set variation analysis (GSVA)

GSVA was performed with the R package “GSVA” (version 1.48.0.) implemented in R version 4.3.0. GSVA was performed to verify whether the genes were correlated with specific genesets. The genesets were downloaded from the Gene Set Enrichment Analysis database (GSEA, https://www.gsea-msigdb.org).

### Statistical analysis

All quantitative data are expressed as mean ± SD. Multiple groups were compared using one-way analysis of variance (one-way ANOVA) with Tukey’s post hoc test. Statistical significance was set at P-value < 0.05. Statistical analyses were performed using the SPSS/Win 13.0 software (SPSS Inc., Chicago, USA).

## Results

### FMD reduces M2 TAM infiltration and synergizes with apatinib to inhibit breast cancer growth in breast cancer mouse model

**Fig. 1:**
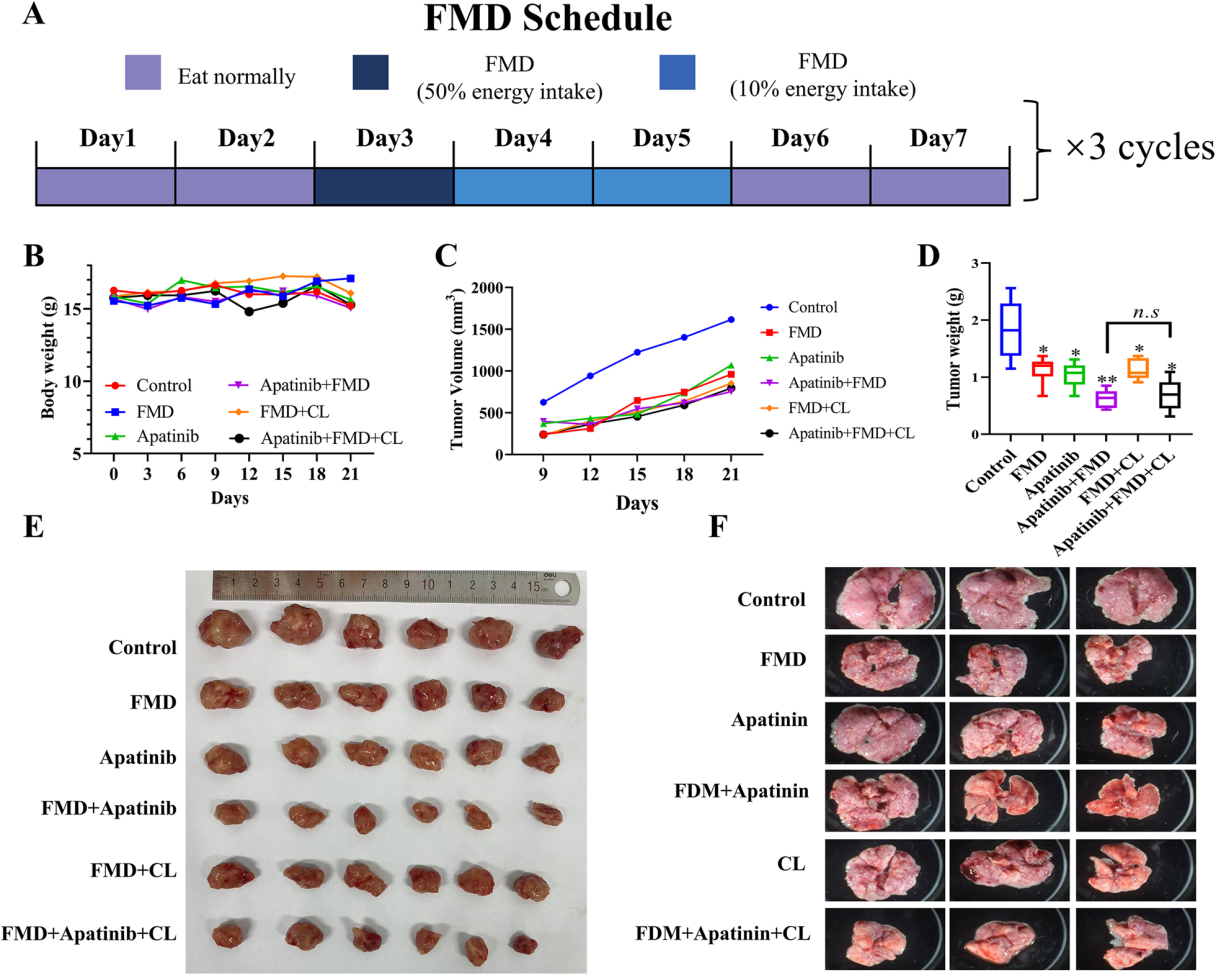
Synergistic effect of FMD and apatinib inhibits the tumor growth and lung metastasis. **A** The schematic diagram of FMD diet plan. **B** The body weights of mice, recorded every 3 days. **C** The tumor volumes of mice, recorded every 3 days. **D** The tumor weight in each group. (n = 6, *P < 0.05, **P < 0.01 vs. control group). **E, F** Representative tumor and lung tissue in each group

In the 4T1-Luc orthotopic syngeneic breast cancer mouse model, mice treated with FMD for a total of three cycles resulted in a decrease in tumor volume as compared to the control group (Fig. 1C–E). Mice treated with a combination of FMD and apatinib demonstrated a reduced tumor volume when compared to those treated with apatinib (Fig. 1C–E). Compared to the control group, lung metastasis was significantly inhibited by apatinib, while it was further potentiated when used in combination with FMD (Fig. 1F). Notably, no significant body weight loss was found (Fig. 1B).

**Fig. 2:**
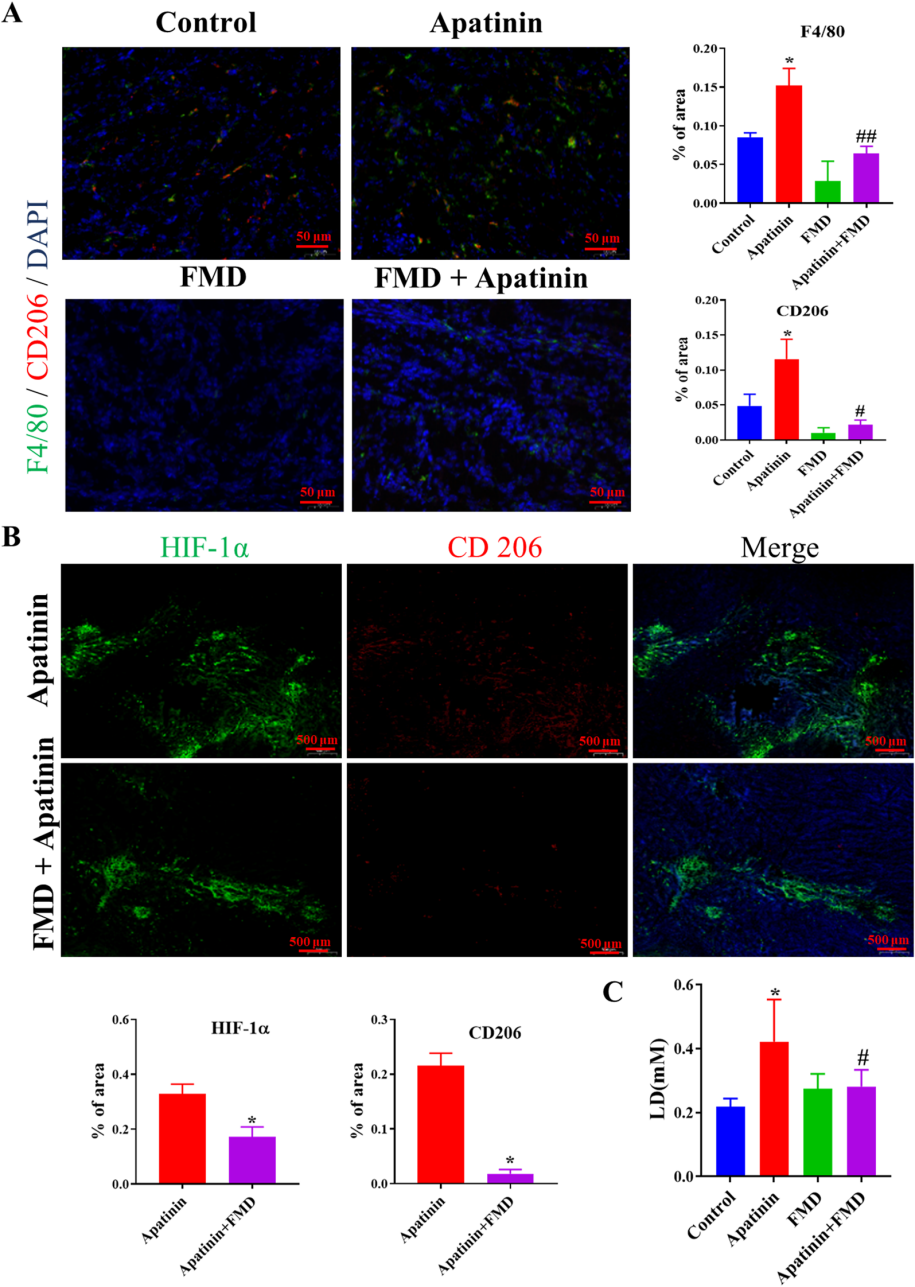
FMD reduces M2 TAMs infiltration in breast cancer mouse model. **A** The expression of F4/80 (green) and CD206 (red) in tumor tissues are detected by immunofluorescence (IF) (magnification, ×40). **B** The expression of HIF-1α (green) and CD206 (red) in tumor tissues are detected by IF (magnification, ×2). **C** The Lactate acid in tumor is detected by lactate acid assay (*P < 0.05 vs. control group, ^#^*P* < 0.05 vs. apatinib group)

On examination of the response to apatinib treatment by immunofluorescence staining of M2 macrophage marker F4/80 and CD 206 antibodies, it was observed that the number of M2 tumor-associated macrophages (TAMs) increased in tumors treated with apatinib as compared to the control group. However, mice treated with FMD exhibited a reduction in TAMs. Furthermore, the infiltration of TAMs induced by apatinib was decreased when used in combination with FMD (Fig. 2A). To confirm that the inhibition of macrophages by FMD was responsible for the observed effects, CL were introduced to deplete macrophages in mice. The results showed that CL did not significantly enhance the anti-tumor effect of apatinib when used in combination with FMD (Fig. 1B–D), thereby suggesting that the FMD-mediated reduction of TAMs synergizes with apatinib to exert its anti-tumor activity.

### FMD inhibits the survival and polarization of hypoxic TAMs by reducing glucose levels

In Fig. 2B, it is demonstrated that TAMs (indicated by the marker CD206) is mainly distributed in hypoxic areas of tumors (indicated by HIF-1α). Our previous studies illustrated the association of tumor-promoting function of hypoxic TAMs with their glucose uptake [11, 19]. Figure 2C showed the reduction of lactic acid (LD), a product of glucose metabolism, by FMD, thus suggestive of FMD inhibiting the survival and polarization of hypoxic TAMs by reducing glucose level.

**Fig. 3:**
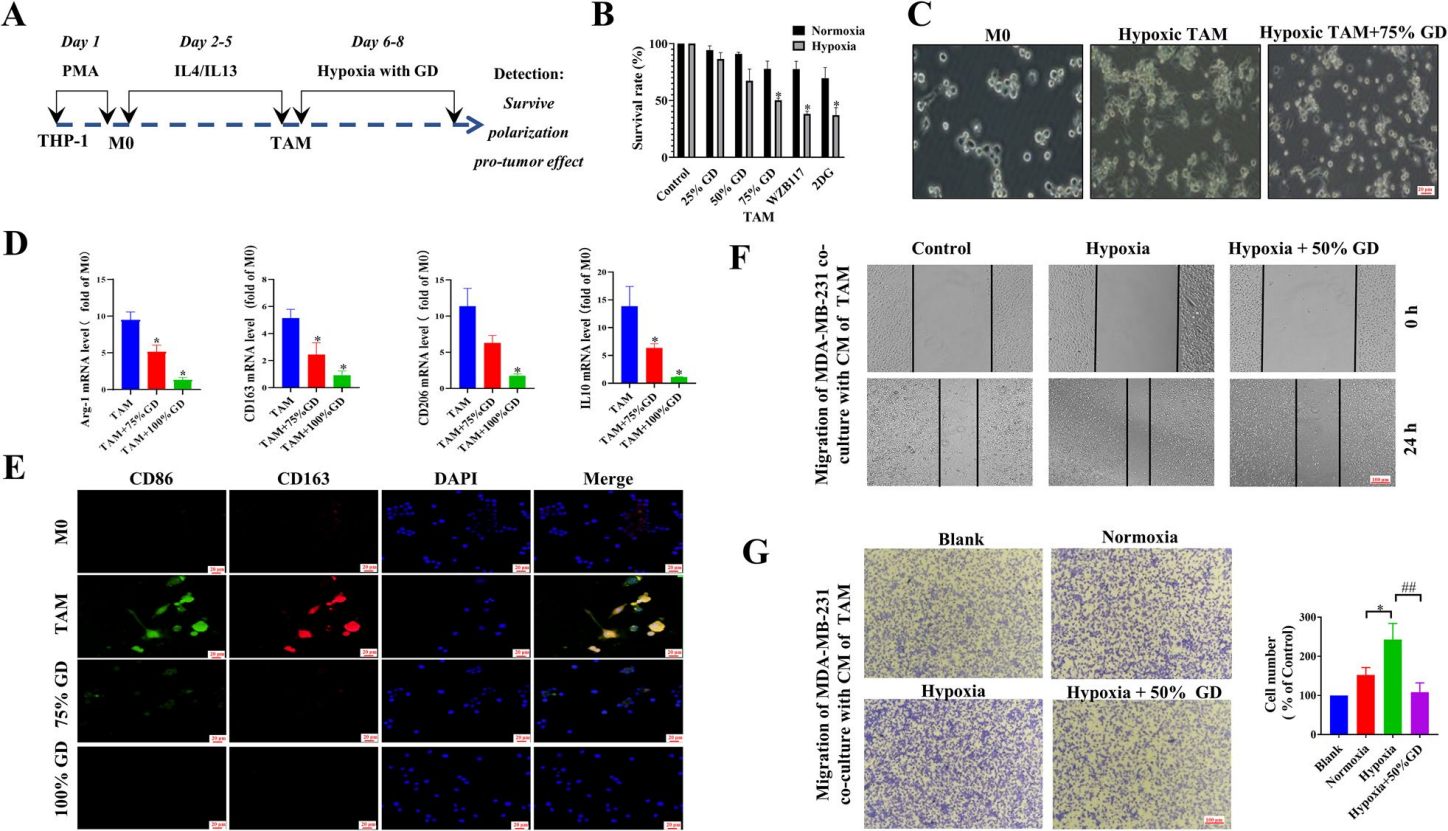
FMD inhibits the survival and polarization of hypoxic TAMs. **A** The schematic diagram of detection of the survival, polarization, tumor-promoting function of hypoxic TAMs after treatment with glucose deficiency. **B** The cell viabilities of glucose-deprived TAMs, and those treated with WZB117 (10 µM), and 2-DG (10 mM) for 72 h are determined by CCK-8 assay. **C** Representative images of TAMs morphology in hypoxia (magnification, ×40). **D** The mRNA expression of Arg1, CD163, CD206, and IL-10 in TAMs under hypoxia are measured by RT-PCR. **E** Expressions of CD 86 and CD 163 of TAMs cells are analyzed by immunofluorescence (magnification, ×40). **F** Migration of MDA-MB-231 cells after co-incubation for 24 h with CM of TAMs is determined using scratch-wound assays. Control group: MDA-MB-231 co-incubation with CM of TAMs; Hypoxia group: MDA-MB-231 co-incubation with CM of hypoxic TAMs; Hypoxia + 50% GD: MDA-MB-231 co-incubation with CM of 50% GD-treated hypoxic TAMs (magnification, ×10). **G** Migration of MDA-MB-231 cells after co-incubation with TAMs for 24 h is determined in Transwell assays. Blank group: MDA-MB-231 incubation without TAMs; Control group: MDA-MB-231 co-incubation with TAMs; Hypoxia group: MDA-MB-231 co-incubation with hypoxic TAMs; Hypoxia + 50% GD: MDA-MB-231 co-incubation with 50% GD-treated hypoxic TAMs (magnification, ×10). Data are presented as the means ± S.E.M. from three separate experiments. *P < 0.05, **P < 0.01, vs. respective TAM group

Further, we chose the various degrees of glucose deprivation (GD) to observe the impact of FMD induced glucose reduction on the hypoxic TAMs in vitro. TAMs used in vitro were obtained from THP-1 cells after treatment with IL-4 and IL-13, and hypoxia model established by cobalt chloride. The CCK-8 assay showed that various degrees of GD and glucose-transporter inhibitor WZB117 diminished the TAMs survival, with a more pronounced effect observed under hypoxia conditions (Fig. 3B). Moreover, treatment with the glycolysis inhibitor 2-deoxy-d-glucose (2-DG) also significantly inhibited the survival of hypoxic TAMs, thus indicating that FMD exerts its inhibitory effect on hypoxic TAMs by suppressing glycolysis.

Notably, M2 TAMs under hypoxia displayed a spindle-shaped or irregular morphology with a slender pseudopodium; however, upon glucose deprivation, the pseudopodium disappeared, and the TAMs became round or oval-shaped (Fig. 3C). This observation suggests that glucose deprivation may has an impact on the polarization of M2 TAMs in hypoxia. The inhibitory effect of glucose deprivation on the polarization of pro-tumor type TAMs were further confirmed by RT-PCR and immunofluorescence (Fig. 3D, E). Furthermore, Transwell and scratch-wound assays showed that glucose deprivation inhibited the ability of hypoxic TAMs to promote cell migration of breast cancer cell line MDA-MB-231 (Fig. 3F, G).

### FMD inhibits pro-tumor migration function of TAMs by mTOR phosphorylation

**Fig. 4:**
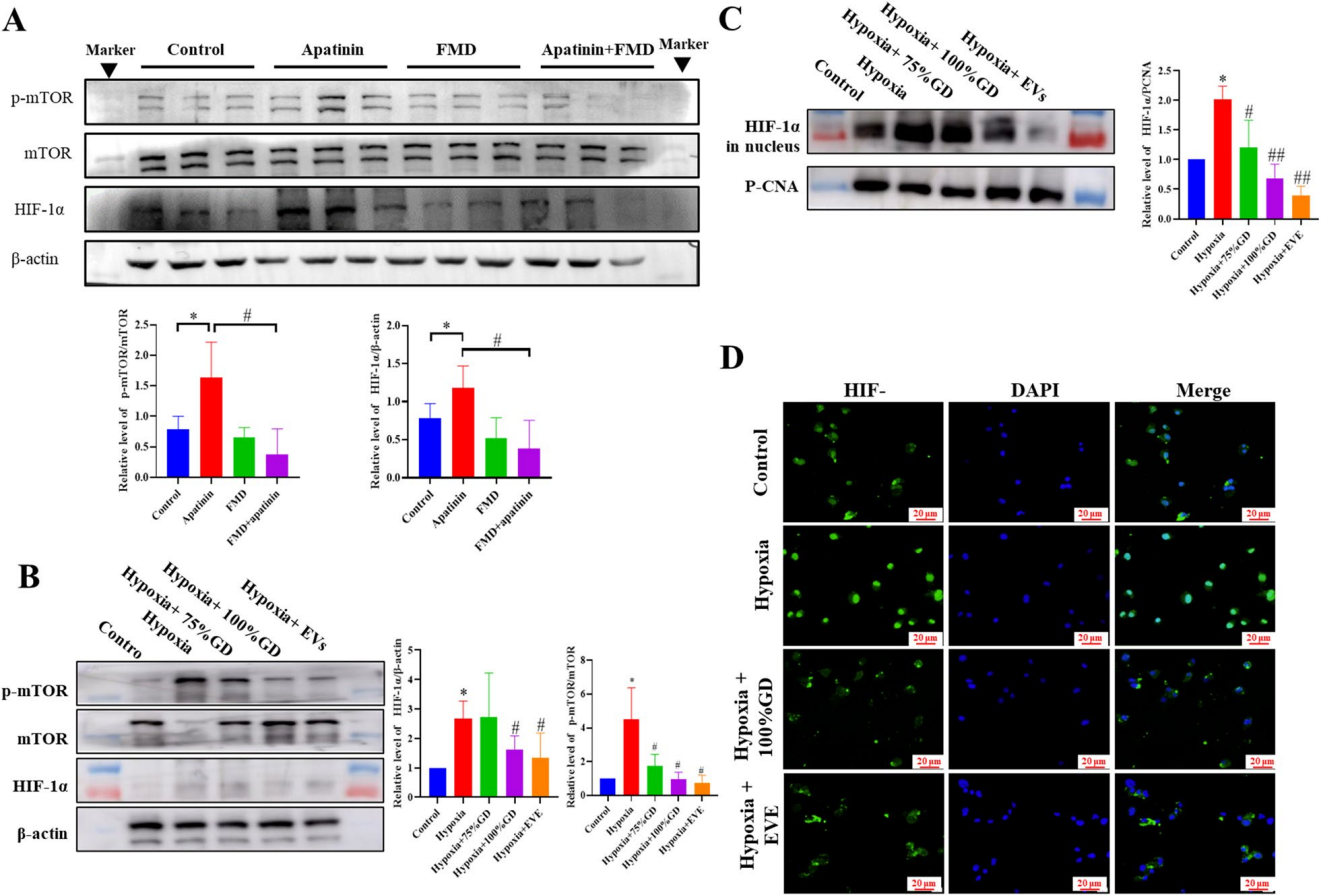
FMD inhibits mTOR phosphorylation in TAMs. **A** mTOR and HIF1-α expressions in tumor tissues are detected using western blotting. **B**, **C** mTOR and HIF1-α in TAMs treated with GD or EVE (100 nM) for 24 h, are detected using western blotting. **D** Immunofluorescence images of HIF-1α expression in hypoxic TAMs (magnification, ×40). Data are presented as the means ± S.E.M. from three separate experiments. *P < 0.05, **P < 0.01, vs. respective control group; ^#^*P* < 0.05 vs. hypoxia group; *n.s* no significant difference

Hypoxia-inducible factor 1-alpha (HIF-1α) and mammalian target of rapamycin (mTOR) are essential regulators of TAMs polarization and glycolysis. Western blotting revealed that apatinib significantly promotes mTOR phosphorylation in mouse tumor tissues, while FMD treatment leads to a significant inhibition of mTOR phosphorylation and HIF-1α expression (Fig. 4A). In the hypoxic TAMs-cell model, GD also inhibited the mTOR phosphorylation and hypoxia-induced nuclear distribution of HIF-1α (Fig. 4B, C). Further, the mTOR inhibitor, everolimus (EVE) inhibited HIF-1α expression in hypoxic TAMs, thus indicating the role of mTOR in the upstream HIF-1α signaling pathway of FMD treatment.

**Fig. 5:**
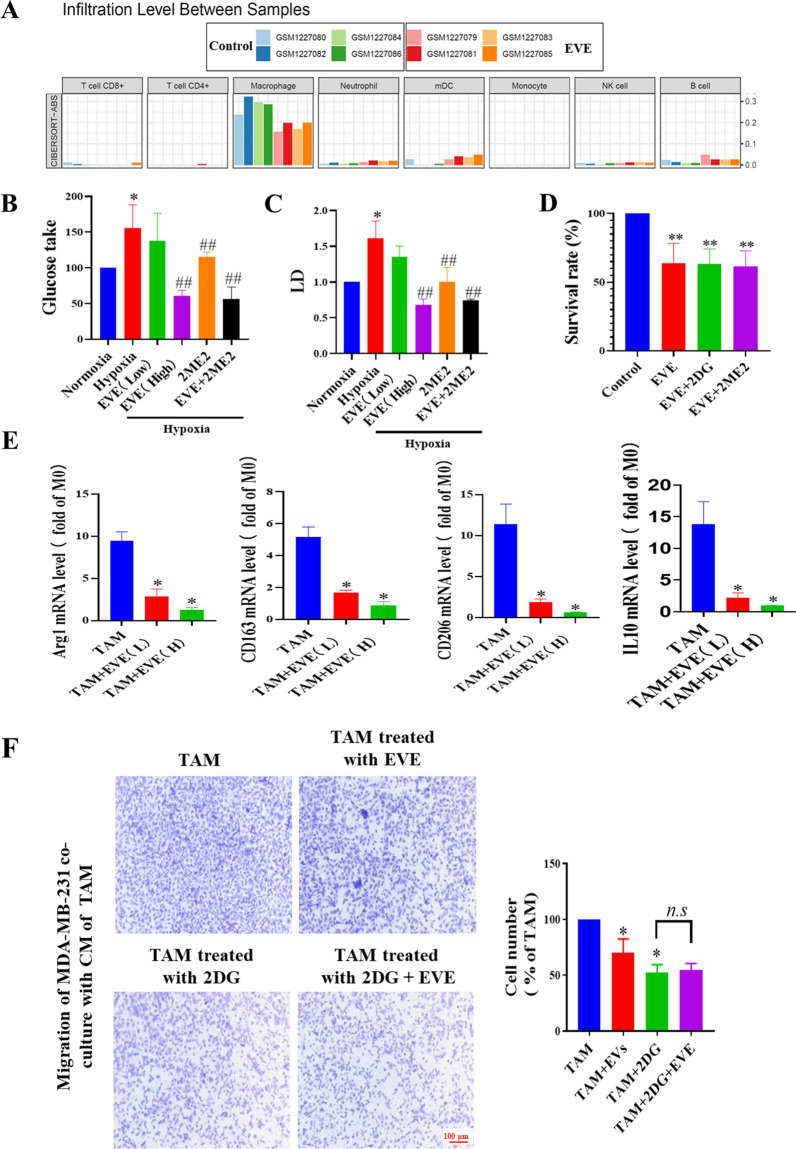
FMD inhibits pro-tumor migration of TAMs by mTOR phosphorylation. **A** Immune cell infiltration is analyzed using data from GEO (dataset GSE50712, Control vs. EVE-treated breast tumor bearing mice). **B** Glucose consumption of TAMs under hypoxia with EVE (100 nM), 2ME2 (10 µM), or 2-DG (10 mM) for 72 h is determined by glucose assay. **C** Lactate acid in the medium supernatant of TAMs under hypoxia with EVE (100 nM), 2ME2 (10 µM), or 2-DG (10 mM) is detected by lactate acid assay after 72 h. **D** The cell viabilities of TAMs under hypoxia with EVE (100 nM), 2ME2 (10 µM), or 2-DG (10 mM) for 72 h are detected by CCK-8 assay. **E** The mRNA expression of Arg1, CD163, CD206, and IL-10 in TAMs treated for 72 h with low (EVE(L), 50 nM) or high doses of EVE (EVE(H), 100 nM), are measured by RT-PCR. **F** Migration of MDA-MB-231 cells after co-incubation with TAMs is determined in Transwell assays (magnification, ×10). Data are presented as the means ± S.E.M. from three separate experiments. *P < 0.05, **P < 0.01, vs. respective control group

Analysis of the Gene Expression Omnibus (GEO) dataset GSE50712 revealed that treatment with EVE led to a reduction in TAMs within tumor tissues (Fig. 5A). Our in vitro data from glucose and lactose detection kits, CCK-8, RT-PCR and transwell assays further confirmed that EVE and HIF-1α inhibitor, 2-Methoxyestradiol (2ME2) inhibited the glucose uptake (Fig. 5B), lactic acid (LD) secretion (Fig. 5C), survival (Fig. 5D), polarization (Fig. 5E) and pro-tumor migration function (Fig. 5F) of TAMs. However, 2-DG and 2ME2 did not significantly enhance the effect of EVE, these results suggest that glucose deprivation inhibits TAMs-mediated tumor metastasis through the mTOR-HIF-1α glycolytic pathway.

### FMD inhibits the secretion of pro-tumor cell migration cytokine CCL8 by TAMs

**Fig. 6:**
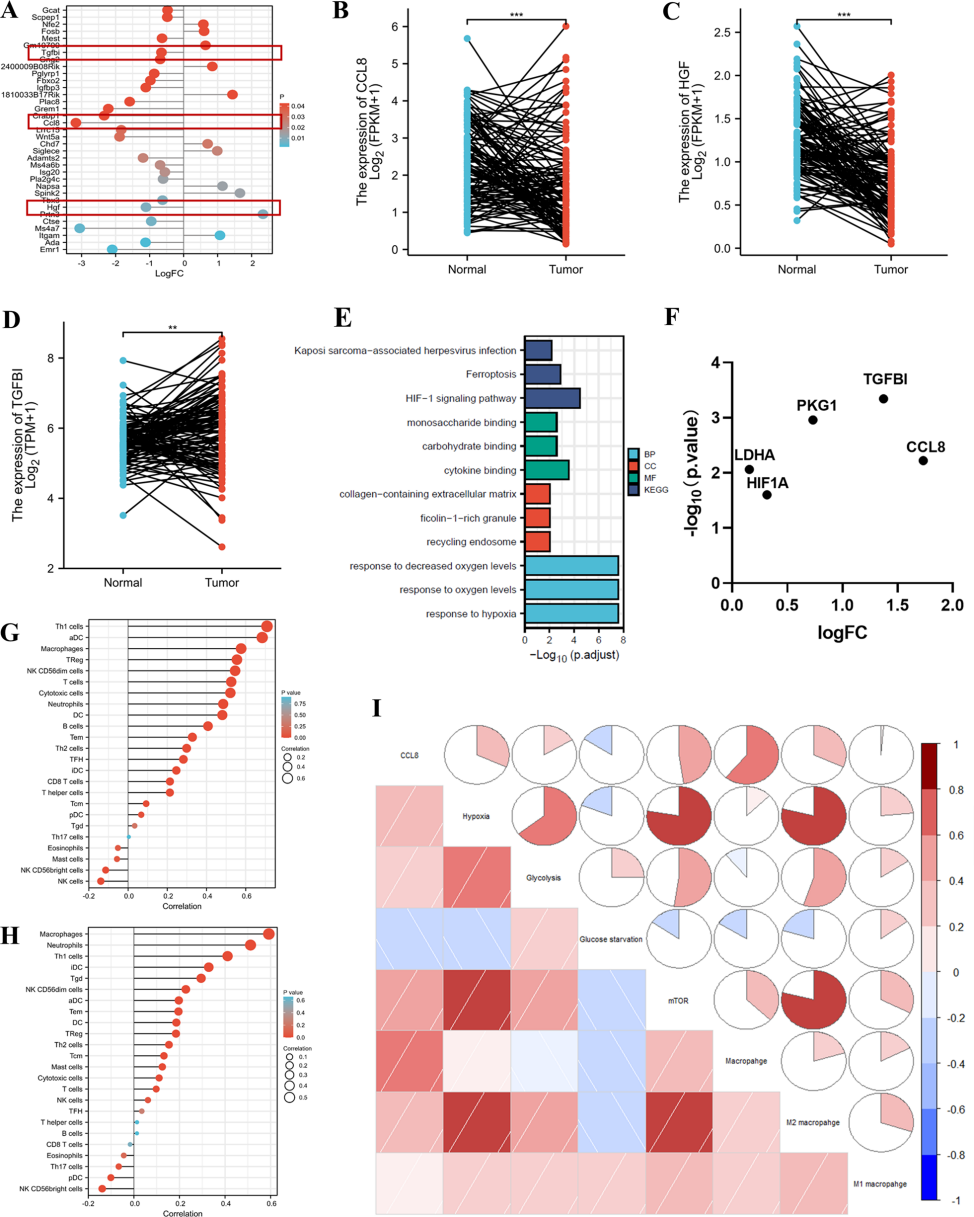
Bioinformatics analysis of the correlation between CCL8 and FMD. **A** Differential gene expression (GEO database, dataset GSE50712, Control vs. EVE-treated breast tumor bearing mice) is determined using GEO2R; the log-fold-change is used to rank the genes. **B**–**D** The expression of CCL8, TGFB1, and HGF in invasive breast cancer samples from the TCGA database. **E** KEGG enrichment analysis for data from the GEO database (dataset GSE37956, control vs. bevacizumab treated glioblastoma xenograft tumor). **F** The expression of CCL8, TGFB1, PKG1, LDHA, and HIF1A in GEO database (dataset GSE37956, control vs. bevacizumab treated glioblastoma xenograft tumor). **G**, **H** Correlation between CCL8, TGFBI and immune cell infiltration in invasive breast cancer samples from the TCGA database. **I** GSVA analysis of correlation between CCL8, hypoxia (Gene set from GESA M34030), glycolysis (Gene set from GESA M5113), glucose starvation (Gene set from GESA M15497), mTOR (Gene set from GESA M2672), macrophage (Gene set from GESA M39708), M2 macrophage (Gene set from GESA M8527), M1 macrophage (Gene set from GESA M6586) in invasive breast cancer samples from the TCGA database

Analysis of the GEO database (dataset GSE50712) revealed that treatment with EVE significantly altered 39 genes (*P* < 0.05) in mouse tumors, with CC chemokine ligand 8 (CCL8), transforming growth factor-beta (TGFBI), and hepatocyte growth factor (HGF) identified as pro-tumor metastasis cytokines (Fig. 6A). Data from The Cancer Genome Atlas (TCGA) database demonstrated upregulation of CCL8 and TGFBI and downregulation of HGF in breast cancer (Fig. 6B–D). Moreover, our previous studies reported the intensification of hypoxia by Bevacumab [11]. Kyoto Encyclopedia of Genes and Genomes (KEGG) analysis of the GEO database (dataset GSE37956) further confirmed that hypoxic signal pathway responds to anti-angiogenic drug Bevacumab, accompanied by upregulation of CCL8, TGFBI and Glycolysis related genes LDHA, PKG1 (Fig. 6E, F). These results imply that hypoxia induces the expression of CCL-8 and TGFBI through mTOR-HIF-1α a while EVE may limit the effect of hypoxia.

Subsequently, we investigated the correlation between CCL8, TGFBI expression levels and immune infiltrates in invasive breast cancer samples from the TCGA database, respectively (Fig. 6G, H). The results indicated that both CCL8 and TGFBI are positively correlated with macrophage infiltration, with CCL8 showing stronger significance. Thus, we further analyzed CCL8 expression patterns in invasive breast cancer samples. As shown in the Fig. 6I, gene set variation analysis (GSVA) showed that CCL8 are positively correlated with gene sets related with hypoxia, glycolysis, mTOR, macrophage, M2 macrophage, negatively correlated with glucose starvation, M1 macrophage in invasive breast cancer samples, respectively. These results imply that hypoxia induces the expression of CCL-8 through mTOR-HIF-1α-mediated glycolytic pathway, which can be suppressed by FMD treatment. In addition, the analysis results of the negative correlation between glucose starvation and hypoxia, mTOR, macrophage, M2 macrophage further supports the in vitro and in vivo experimental results reported in this study.

**Fig. 7:**
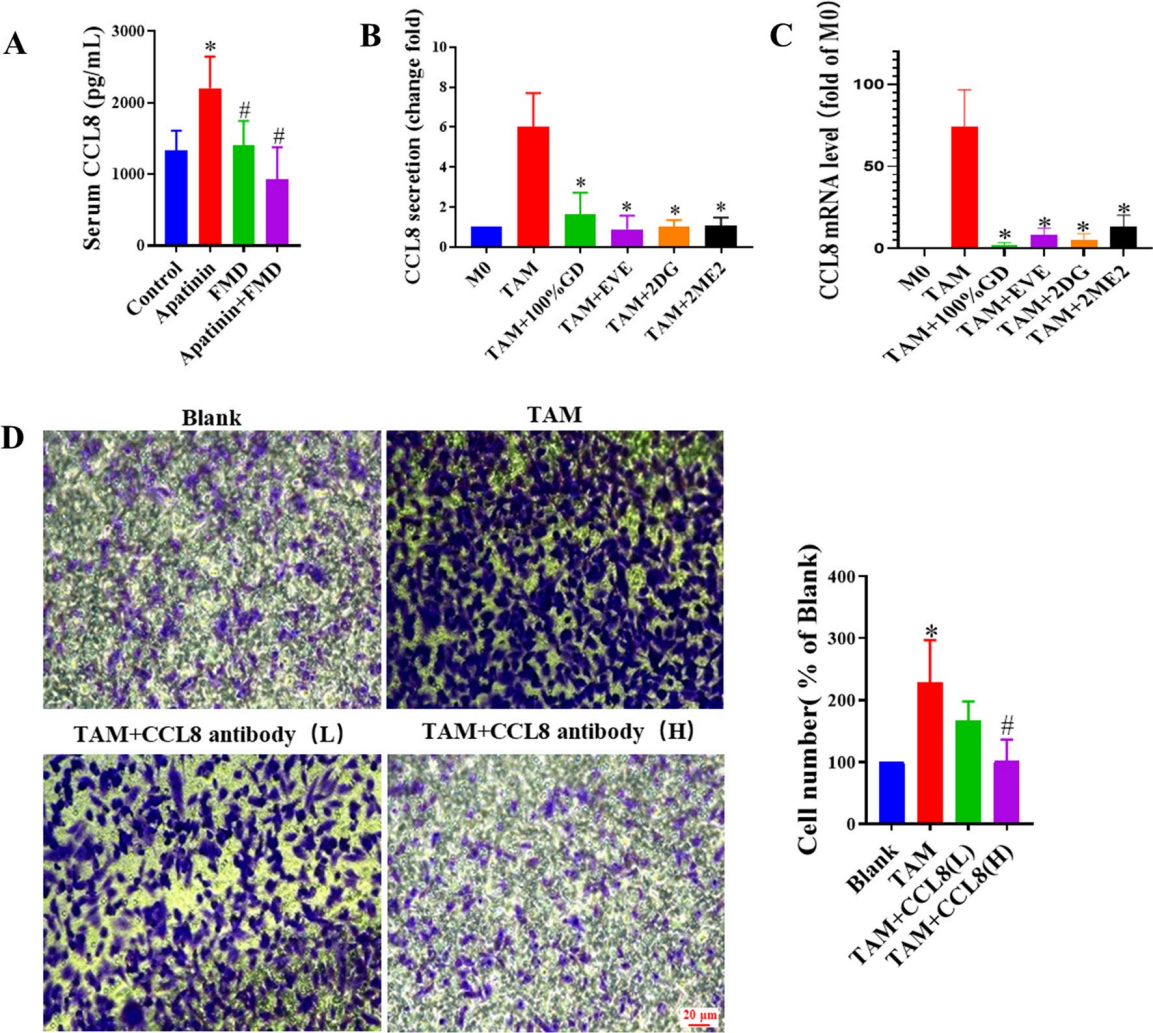
FMD inhibits the secretion of CCL8 by TAMs.** A** Serum CCL8 level in mice is detected by ELISA (*P < 0.05, vs. respective control group, ^#^*P* < 0.05, vs. respective TAM group). **B** CCL8 secretion from TAMs treated for 24 h with GD or EVE (100 nM) or 2-DG (10 mM), or 2ME2 (10 µM) is determined by ELISA (*P < 0.05, vs. respective TAM group). **C** CCL8 mRNA levels in TAMs are determined by RT-PCR (*P < 0.05, vs. respective TAM group). **D** Migration of MDA-MB-231 cells, after co-incubation with TAMs and low (L) or high doses (H) of CCL8 antibody, is determined in a transwell assay (magnification, ×40)

Finally, we further clarified the role of mTOR-HIF-1α-CCL8 in pro-tumor effect by TAMs through in vitro and in vivo experiments. In the breast cancer mouse model, FMD treatment significantly reduced the increase of serum CCL8 of apatinib group (Fig. 7A). In the in vitro cell model, RT-PCR and ELISA results showed that hypoxia-induced mRNA expression and secretion of CCL8 in TAMs were attenuated by GD, EVE, 2ME2, and 2-DG (Fig. 7B, C). Further, neutralizing antibodies targeting CCL8 inhibited the pro-tumor migratory effect of hypoxic TAMs. Collectively, these results suggest that FMD inhibits the secretion of pro-tumor cell migration cytokine CCL8 by TAMs induced by hypoxia via mTOR-HIF-1α-glycolysis pathway.

## Discussion

Preclinical and clinical trials showed that fasting or FMD can reduce the glucose level in tumor tissue [6, 20]. Notably, a recent study has identified TAMs as the primary source of glucose consumption in tumors, surpassing even that of tumor cells [21]. These studies suggest a significant impact of FMD on TAMs. Our previous studies revealed that inhibiting glucose uptake impairs the survival and tumor-promoting function of hypoxic TAMs [11, 19], suggesting that FMD may also restrain the survival and tumor-promoting function of hypoxic TAMs. We further confirmed hypothesis through both in vivo and in vitro in this study. FMD significantly reduced the number of M2 TAMs and attenuated tumor development in vivo, while our in vitro findings showed that glucose deprivation inhibited the survival and pro-tumor migration function of hypoxic TAMs, with a greater inhibitory effect observed under hypoxic conditions rather than normoxic conditions. This indicated the potential cytotoxic effects of FMD on hypoxic TAMs.

The safety and feasibility of FMD have been confirmed in preclinical studies and clinical trials [22], indicating its potential in combination with anti-tumor therapy. Investigating the effects of FMD on TAMs, along with their role in tumor development, drug resistance, and tumor microenvironment shaping, will provide valuable insights into developing combination strategies. Previous studies have demonstrated that anti-angiogenic drugs promote tumor metastasis in a mouse model of metastatic breast cancer, while TAMs induced by hypoxia are implicated as the underlying mechanism [10–12]. The combination of anti-angiogenic drug, sorafenib, and macrophage depletion agent, CL, synergistically inhibited angiogenesis and lung metastasis [12]. To the best of our knowledge, this is the first in vivo study to report the augmentation of the anti-tumor effect of anti-angiogenic drugs by FMD, facilitated by the inhibition of hypoxic TAMs.

Studies have shown that hypoxia upregulates the TAMs-derived cytokines such as vascular endothelial growth factor (VEGF), TGF-β, CCL18 and IL-6, which were involved in tumor progression and metastasis [23]. CCL8, another cytokine produced by TAMs, has been shown to promote tumor invasion and stemness [24, 25]. In our current investigation, we found that the expression of CCL8 in TAMs is induced by hypoxia and the downstream HIF-1α signaling pathway. This finding is consistent with a prior study where HIF-1α-induced zinc finger E-box-binding homeobox 1 led to increased CCL8 expression [26].

To summarize, our findings suggest that FMD can inhibit the pro-tumor effect of hypoxic TAMs and enhance the anti-tumor effect of anti-angiogenic drugs in mice breast cancer mice model. This effect is partially mediated by the downregulation of CCL8 expression and secretion by the mTOR-HIF-1α signaling pathway. Our in vitro and in vivo investigations support the potential of FMD as a therapeutic approach for targeting TAMs and improving the effectiveness of anti-angiogenic strategies. Moreover, our previous research has demonstrated that inhibiting the uptake of glucose of TAMs can also attenuate their pro-angiogenic properties. This suggests that FMD may attenuate the pro-angiogenesis function of TAM and enhance the anti-angiogenic effect of anti-angiogenic drugs.

## Data Availability

The authors declare that the data supporting the findings of this study are available within the paper.

## Abbreviations

2DG: 2-Deoxy-d-glucose
AAT: Anti-angiogenic therapy
CCL8: CC chemokine ligand 8
CL: Clodronate liposomal
CM: Conditioned medium
EVE: Everolimus
FMD: Fasting-mimicking diet
GD: Glucose deficiency
HIF-1α: Hypoxia inducible factor-1α
mTOR: Mammalian target of rapamycin
NF-κB: Nuclear factor kappa-B
PI3K: Phosphatidylinositol 3-kinase
TAMs: Tumor-associated macrophages
TGF: Transforming growth factor
VEGFA: Vascular endothelial growth factor A
